# Multistate study on suicide risk reduction and improving mental well-being among school and college students in India – an implementation research study protocol

**DOI:** 10.3389/fpubh.2025.1708246

**Published:** 2025-12-08

**Authors:** Seema Mehrotra, Chetna Duggal, Neeti Rustagi, Ramdas Sarjerao Ransing, Pillaveetil Sathyadas Indu, Satabdi Chakraborty, Tarun Mene, Pragya Sharma, Deepshikha Prasad, Shankar Prinja, Pulkit Verma, Rajinder K. Dhamija, Kanak Kataria, Manjula Munivenkatappa, Navratan Suthar, Thekkethayyil Viswanathan Anilkumar, Neha Dahiya, Ashoo Grover

**Affiliations:** 1 Department of Clinical Psychology, National Institute of Mental Health and Neurosciences, Bengaluru, India; 2 School of Human Ecology, Tata Institute of Social Sciences, Mumbai, India; 3 Department of Community Medicine and Family Medicine, All India Institute of Medical Sciences, Jodhpur, India; 4 Department of Psychiatry, All India Institute of Medical Sciences, Guwahati, India; 5 Kasaragod Medical College Hospital, Kasaragod, India; 6 Department of Psychiatric Social Work, Institute of Human Behaviour and Allied Sciences, New Delhi, India; 7 Division of Informatics and Data Center, Indian Council Medical Research (ICMR), Headquarters, New Delhi, India; 8 Department of Community Medicine, Maulana Azad Medical College, New Delhi, India; 9 Division of Delivery/Implementation Research, Indian Council of Medical Research, New Delhi, India; 10 Department of Community Medicine and School of Public Health, Post Graduate Institute of Medical Education and Research, Chandigarh, India; 11 Department of Psychiatry, Government Medical College Kollam, Kollam, India

**Keywords:** implementation science, suicide prevention, students, mental health, young adult, india, help-seeking behavior

## Abstract

Suicide remains a crucial public health concern in India, especially among students and young adults. The growing academic pressure, social stigma, and lack of accessible mental health services contribute to the growing crisis and suicide rates in India. The study aims to develop an implementation model for educational institutions for reducing risk of suicide behavior (perceived stress and depressive symptoms) and enhance help-seeking behavior. This multi-sectoral implementation model is grounded in the WHO’s recommendation for youth suicide prevention and UMMEED guidelines. The study will adopt a concurrent mixed methods design, incorporating both quantitative and qualitative aspects that would be captured simultaneously. A formative research phase will be initially conducted before taking on the main study and it will be guided by the Updated Consolidated Framework for Implementation Research (CFIR). Additionally, the evaluation of the implementation process will be structured using the RE-AIM framework. The study participants will be school and pre-university students, and college students The expected outcome will be achieving reduction of suicide risk behaviors among school and college students in selected districts. The key barriers and facilitators identified through this implementation research will inform recommendations for the potential nation-wide scale up of strategies aimed suicide prevention strategies at educational institutes.

**Clinical trial registration:**

ClinicalTrials.gov, CTRI/2024/08/072027.

## Introduction

India has the highest burden of global suicide deaths (1) with a suicide rate of 12.69 in 2019 for both male and female (2). India’s proportion in the global suicide deaths increased from 27.3% in 1990 to 36.5% in 2019 among women and girls and from 16.7% in 1990 to 20.9% in 2019 among men and boys (1). According to WHO, the crude suicide rate in the age-group 15–29 years (per 100,000 population for both sexes) for India was recorded as 15.721 and is one of the leading causes of death among youth (1). Considering the current trends, it may be difficult for India to achieve the Sustainable Development Goal (SDG) 2030 target of reducing the age-standardized suicide death rate (ASDR; 12.1 per 100,000 in 2019) by a third (1).

The student population comprises 7.6% of deaths due to suicide (3). There has been a 25% increase in the socio-environmental and psychological risk factors among school and college students since COVID-19 pandemic, while there is a decrease in the protective factors (4). The alarming suicide rate among the students/youth (3) and the lack of accessible interventions and services in school and colleges provides the requisite thrust for evolving systems for mental health promotion in national educational settings.

Suicides and suicidal behavior are frequently the result of dynamic and complex interactions between various bio-psycho-social factors (5). Therefore, addressing these factors is vital for adapting and implementing any evidence-based models for suicide prevention.

The biological risk factors for suicides or suicidal behavior are mainly age (15–24 years age group has highest suicide rate in India), gender (female>male), biomarkers (e.g., dysfunctional brain circuitry, altered serotonin level), genetic factors, and presence of mental disorders/substance use (6). Importantly when such disorders oc-occur, the risk is magnified, with comorbidity serving as a critical determinant of suicidality (7). In contrast to the risk factors, several protective factors have been identified. These include problem-solving skills, coping skills, access to physical and mental health care, social connectedness, and healthy lifestyles are some of the protective factors (5).

The psychological risk factors are broadly organized into affective, cognitive, and social processes. Affective processes such as low self-esteem, worthlessness, neuroticism, hopelessness, difficulty in identification of emotions, ineffective emotional regulations, result into persistent and elevated risk of suicidal behavior in youth (6). Also, the cognitive processes such as impulsivity-aggression traits, selective-information processing are the predictors of suicidality among youths. The social processes such as loneliness (often mediated by other psychopathologies such as depression, anxiety) are associated with suicidality among youth (5).

In addition, recent advances in the literature suggest that psychiatric comorbidity further exacerbates suicide risk. Anxiety and depressive symptoms are significant predictors for the onset of suicidal ideaton and attempts among youth (7). Furthermore, individuals with multiple co-occurring mental health conditions exhibit substantially higher vulnerability to suicidal behaviors as compared to those with a single diagnosis (8). These findings underscore the compounded risks faced by individuals, particularly youth, who struggle with multiple mental health issues.

There is substantial evidence highlighting socio-environmental risk factors associated with youth suicide. These include child/adolescent maltreatment (any form of child abuse-physical, sexual or emotional), bullying (including cyberbullying) (9), peer and media influence, media reporting, drifting (getting disconnected from one’s support system-e.g. school, family), family constellation (disrupted family, parental loss may be due to death or divorce) (10), exam related factors (e.g., pressure to perform, failure, anxiety), minorities/ethnicity/caste-based discriminations, availability of lethal means, gender identity and sexual orientation, perceived stigma and availability of mental health resources (5).

Given the complex interplay of the risk and protective factors, suicide risk prevention requires multi-sectoral and multi-level approaches that consider evidence-based, universal, selective, and targeted strategies. Existing literature on suicide prevention, including studies conducted in educational settings, highlight a significant lack of implementation research in this field, particularly regarding the application of implementation frameworks in low and middle-income countries (LMICs). There is limited evidence supporting sucicide prevention programs in institutions of higher education and universities, underscoring the need to strengthen the evidence base in LMICs. A recent scoping review of 30 years of sucicide prevention in university students identified major gaps in efficacy, effectiveness and cost-effectiveness (11, 12). WHO also emphasizes that youth suicide prevention remains under-prioirtized in many LMICs, with limited integration into educational systems (13). This gap highlights the urgent need for contextually and culturally relevant implementation research within educational settings in LMICs like India.

The National Suicide Prevention Strategy (NSPS) of India aims at reducing suicide mortality by 10% by establishing effective surveillance mechanisms (by 2025) (14), integration of suicide prevention services via District Mental Health Programme in all districts (by 2027) (14), and integrating a mental wellbeing curriculum in all educational institutions (by 2030) (14). Additional objectives of the NSPS aim at strengthening leadership, fostering partnerships, and building institutional capacity at national level. The strategy also aims at capacity building at the level of health services for provision of suicide prevention services, promote community resilience and societal support, reducing stigma attached to suicidal behavior and advancing surveillance of suicide and evidence-based research (14).

The alarming suicide statistics among youth (3), an evidence-base on effectiveness of comprehensive multi-component intervention approaches including recommendations on implementation strategies under NSPS, highlight the urgent need for developing evidence-based suicide prevention model/s in educational campuses that are socio-culturally appropriate, acceptable, scalable and sustainable in resource-constrained settings.

In the present study interventions are specifically selected using the expert consensus method after considering their cost-effectiveness, universal approach, feasibility in non-health care settings (school-less stigmatizing approach), national suicide prevention strategies recommended by the Ministry of Health and Family Welfare, UMMEED guidelines of the Ministry of Education, and WHO Live Life Framework for Suicide Prevention. These intervention components will be linked to each other using the Sakashita model for suicide prevention (15) based on findings of formative phase of research.

### Aims and objectives

The study aims to co-develop an implementation model for educational institutions for reducing risk of suicide behavior (perceived stress and depressive symptoms) and enhance help-seeking behavior through formative research.

The primary objectives are to (a) develop an implementation model for educational institutions for reducing risks of suicide behavior (perceived stress, depressive symptoms) and enhance help seeeking behavior through formative research, (b) optimize the model through repetitive cycles of implementation, evaluation and refinement and (c) scale and evaluate the final implementation model.

## Methods and analysis

### Study design

The study will adopt a concurrent mixed methods design, incorporating both quantitative and qualitative aspects that would be captured simultaneously. A formative research phase will be initially conducted before taking on the main study and it will be guided by the Updated Consolidated Framework for Implementation Research (CFIR) (16). Additionally, the evaluation of the implementation process will be structured using the RE-AIM framework (17).

### Study setting

This study will be conducted in selected districts of eight states and union territories in India. The states and union territories are self-governing administrative divisions, and each of them has a state government. The administrative authority in these regions is shared among the state government and the central government. The study in each state and union territory shall be overseen by investigators belonging to a local institution. These states, union territories and institutions offering oversight include Andaman & Nicobar Islands (Andaman and Nicobar Islands Institute of Medical Sciences, Sri Vijaya Puram), Arunachal Pradesh (Rajiv Gandhi University, Papum Pare), Assam [All India Institute of Medical Sciences (AIIMS), Guwahati], Karnataka [National Institute of Mental Health and Neuro Sciences (NIMHANS), Bengaluru], Kerala (Government Medical College, Kollam), Maharashtra [Tata Institute of Social Sciences (TISS), Mumbai], New Delhi [Institute of Health Behaviour and Allied Sciences (IHBAS)], and Rajasthan [All India Institute of Medical Sciences (AIIMS), Jodhpur].

Within each state and union territory, one district has been selected for the study except for states of Arunachal Pradesh, Assam, Karnataka and Kerala. These include South Andaman (Andaman & Nicobar Islands); Dibang valley, Lower Dibang valley, Lohit, Papumpare, Itanagar capital complex, and Lower Subansiri (Arunachal Pradesh); Kamrup Urban/Rural (Assam); East Delhi (New Delhi); Bengaluru urban and rural (Karnataka); Thiruvananthapuram and Kollam (Kerala); Thane (Maharashtra) and Jodhpur (Rajasthan). These districts have been identified based on the reported suicide rate (3) and in consultation with respective state education and health departments. Intervention will be implemented at educational institutes in the selected districts. Educational institutes in all spheres including government aided, government-tribal aided and private funded shall be included in the study. The coordinating site would be the Indian Council for Medical Research (ICMR), Headquarters, New Delhi. The estimated time of completion of data collection will be December 2026, and the results are expected in March 2027.

### Study clusters

The educational institutes in selected districts will serve as the clusters for the study. The institutes with school and/or pre-university students (grades 9 to 12) or college students (graduate and postgraduate levels, including professional courses; excluding doctoral) will be eligible to participate.

### Study population

The target population will be school, pre-university and college students across educational institutes in selected districts across the eight states and union territories. The educational institutes exclusively catering to adolescents with special needs (e.g., intellectual and developmental disability or any other) will be excluded. However, such students if enrolled in the institutes selected in the study, they shall be able to access the interventions offered as part of the study.

### Inclusion criteria

The institutes with school and/or pre-university students (grades 9 to 12) and college students (graduate and postgraduate level including professional courses) willing to participate in the study and provide consent shall be eligible for inclusion in the study.

### Exclusion criteria

Students enrolled in doctoral programs and seeking education in distance or online mode and those pursuing certificate courses of less than 1 year duration will be excluded from this study. Additionally, individuals refusing to consent or not interested in participating in the study.

### Sample size and sampling across sites

Each of the eight states/union territories participating in this study will choose 1 or a maximum of 2 districts for implementation. (except in Arunachal Pradesh where more than 1 district will be included to complete the sample size). The choice of the district/s will be based on a host of factors like number and diverse types of educational institutes, feasibility issues, as per consultation with the government officials and any other relevant factors such as high suicide rates (wherever available).

### Sample size for individual level effectiveness

The sample size for youth beneficiaries is calculated to detect a 0.20 effect size{Anticipated per SD effect size(reduction in depression score) = 0.2 (Baseline mean = 8.5; Endline mean = 7.5, SD = 5.0; ES/SD = 1/5 = 0.2)} in mean on the PHQ-9 score in a group (90% power, 2-sided 5% significance level), assuming a correlation of 0.5 between T1 (Baseline) and T4 scores (3 measurements, 6, 12 and 18 months from baseline), and non-response rate of 10%, The required sample size will be 400 students per strata (i.e., 400 for schools, and 400 for UG/PG college) of selected educational institutes.

Average size of the class per stratum could be used to determine the number of classes to be randomly selected from each stratum (e.g., if the average class size for 9 th and 10 th grade is 40, then (400/40 = 10)10 classes would be required to get the sample of 400 in this stratum). The unit for sampling at the college could be batch/year of study and a similar approach could be used to determine the number of units to be followed up.

### Sample size for clusters

The unit of analysis in this multicentric study being cluster, the sample size for each site has been determined based on the presumption: anticipated intervention package adoption rate of 80%, a desired absolute precision of 10% and a 95% level of confidence. Based on these parameters each study site requires 64 (±5) institutes. Consequently, a total of 472–552 educational institutes will be included across the eight states and union territories.

### Sampling method

We will be using a multistage sampling approach that will be adopted to ensure representation across the state and union territory. Each study site will prepare the list of educational institutes (clusters) for selected district(s) and stratify it according to the type of educational institutes (schools/pre-university and colleges) to select at least 64 clusters as per population proportionate sampling. If a selected cluster refuses to participate or withdraw at an early stage, a new cluster will be selected from the remaining clusters.

### Intervention components

The intervention components of the research study include tools for assessment of suicide risk (perceived stress and depressive symptoms), management and student education and engagement for mental health awareness across selected education institutions (Table 1).

**Table 1:** Snapshot of intervention components and their implementation.

S. No.	Intervention	Setting of implementations	Delivery agent	Final delivery format	Training
1.	Leader engagement for mental health first response	Schools and colleges	Principals/Vice-Chancellors/Administrative head/leader (1 from each educational institution selected)	Coordinate the program in their institution by giving due permissions and nominating stakeholders Creating a referral system or SOP for students at risk.	Half day workshop mode A minimum of 2 workshops – one for orientation and awareness building and another for building SOPs and referral systems. Follow up meetings/consultations one on one or in group as needed
1.	Youth engagement for mental health advocacy and peer support	Schools, Pre university and colleges	Youth champions under guidance of teacher-facilitator (at least 2 students per section/class)	Conducting Campaigns and other whole campus activities as per action plans drawn during the training program for awareness & destigmatization Engaging in supportive interactions with peers, particularly peers who may be experiencing significant stress/distress and motivating them to seek further support as per need	2-day workshop mode
2.	Gatekeeper training	Schools, pre university and colleges	Teachers, wardens and counselors * At least 30% of teachers who cater to the target population of youth. The teacher selection should attempt to ensure that there is one gatekeeper-teacher per section	One-to one interactions with distressed youth/youth at risk (those who approach the teacher and those whom the gatekeeper proactively approaches as well as those referred by youth champions) Referral to counselor/other services as per the need and in keeping with the established protocol and follow up	1-day workshop mode (Post training: review and support meetings once in 3-months with research team for the first year and once in 6 months subsequently among gatekeepers)
3.	Preventive and promotive self-help Modules	Schools and colleges	Predominantly self-driven accessed by youth as per their need Note: Where resources are available, counselors/inclined teachers may choose to deliver some of the modules in workshop formats	Access may be provided in a variety of formats based on suitability and feasibility. e.g. printed booklets, videos available in online portal and app-based modules etc. accessible in library/reading rooms/hostel common rooms/wellness centers/counselor office	Counselors/teachers who opt to deliver some of the modules themselves can use the videos/training modules for self-learning

#### Leader engagement

The involvement of school/college leaders is of paramount importance in development, acceptability and implementation of standard operating procedures to provide effective mental health, suicide prevention and crisis intervention care for students (18). This initiative aims to establish standardized procedures that facilitate efficient mental health, suicide prevention, and crisis intervention care for students within educational institutions. By engaging leaders in this process, it ensures that protocols align with the unique dynamics of the school or college environment and the specific needs of the student population, ultimately fostering a safer environment and integrating them with institutes’ broader policies and culture and more supportive mental health ecosystem.

Identifying key stakeholders for community engagement in suicide prevention is an established best practice for effective suicide prevention efforts (19). Second, buy-in from school/college leaders aids in the effective delivery of suicide prevention programs through coordination, resource allocation, monitoring, and supervision. Awareness raising and sensitization about mental health and suicide is a necessary first step in ensuring effective community engagement for suicide prevention (19). Guidelines for administrators on suicide prevention in schools spotlight the need for their involvement in establishing crisis response protocols, designating responsibilities to staff for the implementation of prevention activities, documenting steps in the prevention process, and coordinating referral pathways (20). These protocols focus not only on mental health promotion but also on early identification, referral and suicide postvention (21). Resource mapping of available mental health resources for students at risk is a critical adjunct to identification and screening efforts (22). In the present study this will entail mental health orientation sessions for principals and administrators, collaborative resource mapping of mental health resources within and beyond educational institutes. Consultative meetings will be conducted to collaboratively develop comprehensive contextually relevant protocols for mental health and suicide first response in schools, including the setting up of crisis response teams where applicable. In addition, workshops would be conducted to develop standard operating protocols for referral pathways and monitoring-supervision frameworks and protocols for suicide prevention strategies in educational institutes.

#### Gatekeeper training for suicide prevention

Gatekeeper training has emerged as a key component in suicide prevention strategies, particularly within educational institutes. It aims to build the capacity of lay persons such as school staff, wardens and students to identify signs of distress, provide initial assistance and facilitate access to professional help to at-risk individuals. It is one of the most extensively used and recommended interventions for suicide prevention across educational settings (23–25). Gatekeeper training aligns with the central principle of early identification and provision of support to those at risk, which is a hallmark of comprehensive suicide prevention approaches (21, 26, 27, 62). and is also recognized as key component under India’s National Suicide Prevention Strategy (14).

Many at-risk students are often reluctant to seek help (28). Therefore, trained teachers, support staff and peers can play pivotal roles in identifying and supporting students at risk. Gatekeeper training can be helpful to address common barriers such as discomfort in carrying out suicide risk assessments through interviews, apprehensions about discussing suicide and fear of exacerbating the situation while offering assistance (29). Gatekeeper training has been shown to improve knowledge, attitudes and skills of gatekeepers (29) as well as enhance referrals and help-seeking among youth (30). Gatekeeper training will consist of a brief, 1-day program, supplemented by supporting materials and tools to guide teachers in their gatekeeping role, including suicide awareness and sensitisation, skills in providing psychosocial support, identifying signs of suicide and related distress, first response guidelines and referral procedures as well as self-care. To ensure ongoing support and effectiveness, review and support meetings will be held for all gatekeepers. These meetings will occur quarterly during the first year, with continued support every 6 months thereafter. These meetings will be facilitated by the research team to provide guidance and reinforce best practices.

#### Youth engagement for mental health advocacy and peer support

Multiple guidelines including the WHO’s Helping Adolescents Thrive Toolkit, offer strategies to promote and protect adolescent mental health while reducing self-harm and other risk behaviors. SAMHSA’s preventing suicide toolkit for high school students, National Suicide Prevention Strategy of India, 2022 as well as available research indicates the need to actively engage and mobilize students/student-volunteers for promoting mental wellbeing and suicide risk reduction (31, 32). These initiatives involve training students to offer basic psychosocial assistance to their peers, recognizing the importance of informal support in promoting help-seeking. Peer support has been shown to lead to improvements in depressive symptoms (33), and in adolescents, the use of an online peer support forum has been associated with lower levels of emotional distress (34). Evidence also demonstrates that while peer support is beneficial as a universal intervention, it can have notably enhanced utility for those at risk or experiencing distress and/or mental health problems; peer support which follows a participatory and empowerment-focused approach is demonstrated by evidence to be an important component of multi-component interventions targeted at youth well-being (35). Youth engagement programs focus on equipping young volunteers and nominees with the knowledge and basic skills of mental health and wellbeing, caring for mental health in self and others, and help seeking for mental health. A pool of students called Youth Champions will be formed and trained in each institute by leveraging existing youth platforms or clubs, or by establishing new groups consisting of youth volunteers. This process will be facilitated through announcements made via various channels or during events that highlight the role of Youth Champions. Hybrid methods of recruitment of youth champions may also be employed to maximize participation. At least two students from each section or class of the target group of students within a given campus will be included as Youth Champions. In parallel, teacher facilitators will be identified, with a minimum of two facilitators per campus, based on the campus size and the number of Youth Champions. Teachers may either self-nominate or be nominated by institutional heads.

Youth Champions and teacher facilitators will undergo a training program, which will include 2-day workshops aimed at enhancing their awareness about mental health, common mental health concerns, obstacles to help seeking, self-care strategies for mental wellbeing and importance of peer support. The youth champions will be guided to plan and conduct mental health campaigns and activities in their respective campuses to spread awareness, destigmatize mental health help seeking and will also serve as basic peer support providers for students whom they may observe to be in distress. This training will equip them with the skills necessary to implement their respective plans with institutional support. Periodic meetings will be held to discuss progress, review outcomes, and plan for the subsequent 6-month period. Additionally, youth champions in colleges who have completed basic peer support training and demonstrate strong help-giving behaviors may be considered for inclusion in gatekeeper training, contingent upon their motivation and other campus-specific factors.

#### Preventive and promotive self-help interventions for youth

Promotive and preventive self-help interventions are evidence-based approaches aimed at enhancing positive mental health, preventing mental health issues, and reducing self-harm and risky behaviors (24). These interventions include modules designed for youth to increase their understanding of mental health, expand their range of psychosocial skills for maintaining well-being, and promoting personal growth. Through the adoption of a knowledge-attitude-practice paradigm, these interventions foster holistic change and growth. Evidence demonstrates that universal interventions (i.e., those available to and accessible by *all* students) often elicit lesser stigma than other interventions and demonstrate enhanced participation and adoption as a result (36). Interventions specifically focusing on enhancing youth awareness of mental health and well-being such as the Youth Aware of Mental Health program (37) have demonstrated efficacy in enhancing mental health literacy, reducing stigma and improving help-seeking (38), and are part of the WHO’s toolkit on Helping Adolescents Thrive (HAT) (24). In addition, digital interventions for youth based on cognitive-behavioral principles show increasing evidence in the treatment of depression and anxiety (39), their acceptability with young people at risk for suicide and in their potential to reduce risk (40). Digital tools offer ease of access, flexibility of use, low resource requirements, and high potential for scalability. Targeted interventions also have the potential to improve help-seeking using the adaptation of established frameworks to the Indian context (41).

Based on these literature evidences a repository of evidence-based self-help modules and tools will be developed by the research team, tailored to be appropriate for school and college settings. These interventions align with the WHO’s comprehensive suicide prevention strategies for increasing help-seeking behavior and promoting social and emotional competencies. To ensure broad accessibility, these materials will be distributed across multiple locations within the educational institutions, including reading rooms, libraries, hostel common rooms, health and wellness centers, counselor’s offices, websites, and mobile apps. Based on resources and preferences in given settings, these could be disseminated as online self-help tools for self-driven use, in person (delivered by a facilitator in the form of periodic workshops), made available as reading materials in the form of booklets and flyers or in a hybrid manner involving some combinations of these modes as appropriate.

**Figure 1:**
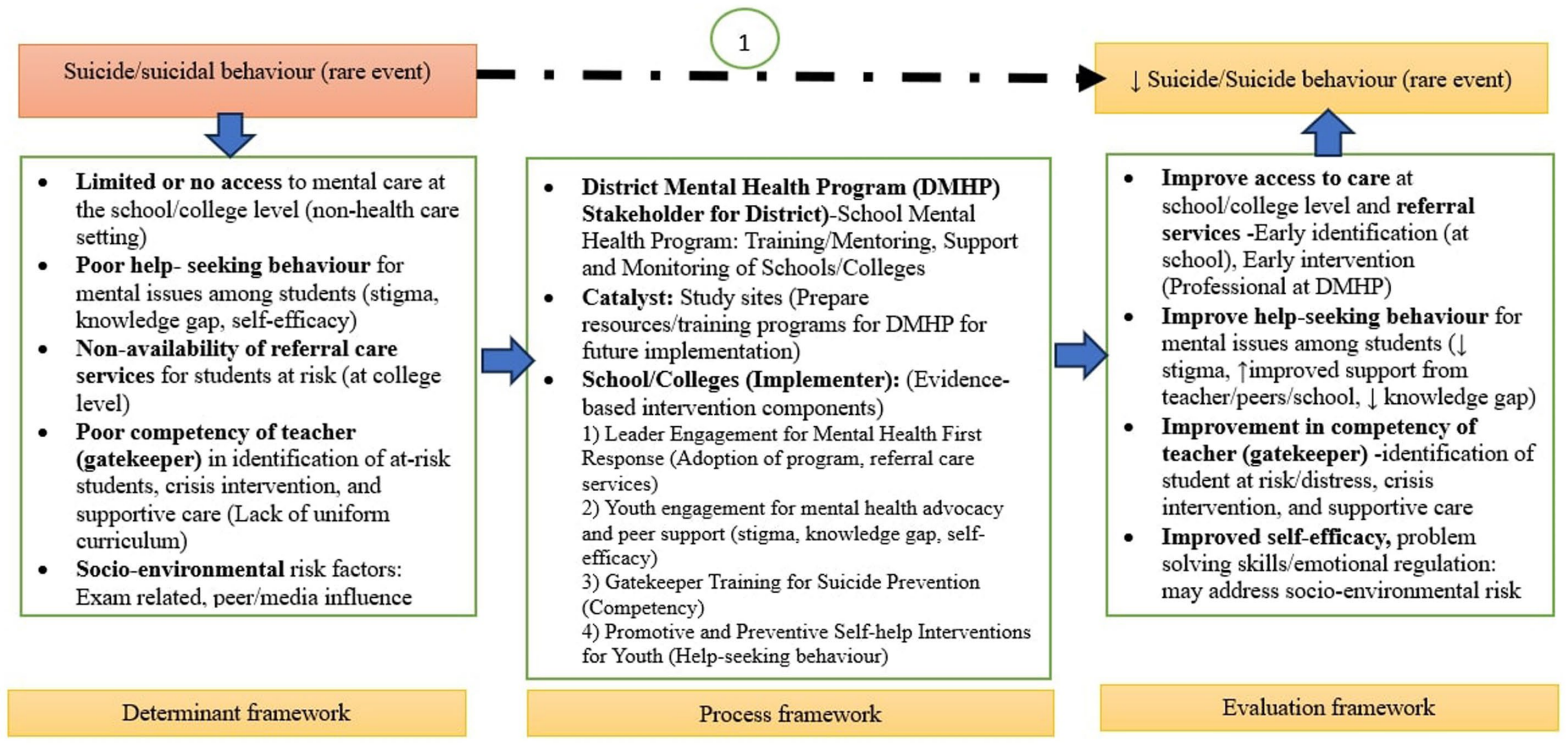
Illustration of implementation model (M0 model) for suicide prevention: Potential mechanism (63).

The availability of these resources will be widely communicated through youth champions, who will play a key role in disseminating information. Additionally, teachers and institutional heads will promote the resources through announcements at large programs or events, as well as through the strategic display of well-designed posters in common areas and the distribution of circulars. This multi-channel dissemination approach will ensure maximum reach and engagement with the self-help resources across the institution (Figure 1).

### Status and timeline of the study

The study has been sanctioned for the duration of 3 years, from 01.03.2024 to 28.02.2027.

- Recruitment and data collection

- Recruitment initiation: participant recruitment is ongoing.
- Data collection completion: Data collection is anticipated to conclude by December 2026.
- Results availability: The final results will be available within three months after the completion of data collection at each site.

### Phases of study implementation

**Figure 2:**
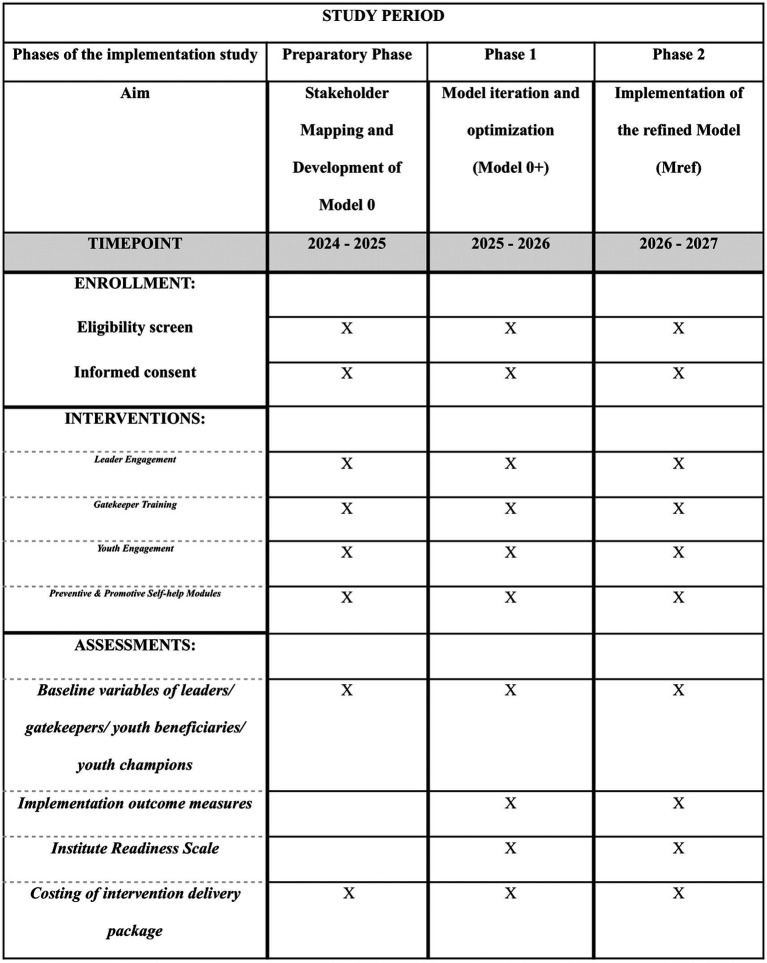
SPIRIT figure of study phases.

The study shall be carried out in three phases including Preparatory Phase, Phase I and Phase II as depicted in Table 2. The spirit figure of the phases is provided in Figure 2.

**Table 2:** Logic model for comprehensive mental well-being package scale up.

Phase	Preparatory phase	Phase 1	Phase 2
Duration (Months)	6	12 months	12–18 months
Key activities	Map and engage all leaders and policy makers (State and district education and health system) to gather support for adoption and implementation of intervention package in educational institutes Conduct workshops in sub sample of selected clusters (4–5 schools) to understand the barriers and enablers influencing adoption and adherence of intervention package	Minimum of 3 to 4 iterative cycles for model optimization with each cycle spanning for 2–3 months in 4–5 clusters of schools. Evaluation of implementation outcomes (Feasibility, utility, costing, sustainability and scalability) at baseline and at end of 2–3 months of implementation for each cluster Identification of barriers to service users -students and service providers-gatekeepers/DMHP	Implementation of Mref model Assessing risks of suicide behavior (perceived stress, depressive symptoms, low help-seeking) at baseline and at atleast at 6, 12 and 18 months Evaluation of implementation outcomes (Feasibility, utility, costing, sustainability and scalability) at baseline and at least at 6, 12 and 18 months
Key outcome	M0 + Model Mapping of stakeholders and resources Onboarding of Clusters Adaptation of measurement tools Developing SOPs for leaders/gatekeepers/youth champions/district mental health professionals	Mref Model Fine tuning of SOPs for leaders/gatekeepers/youth champions/district mental health professionals	Implementation of Mref. model in reducing suicide risk behavior in educational institutes

### Preparatory phase

**Figure 3:**
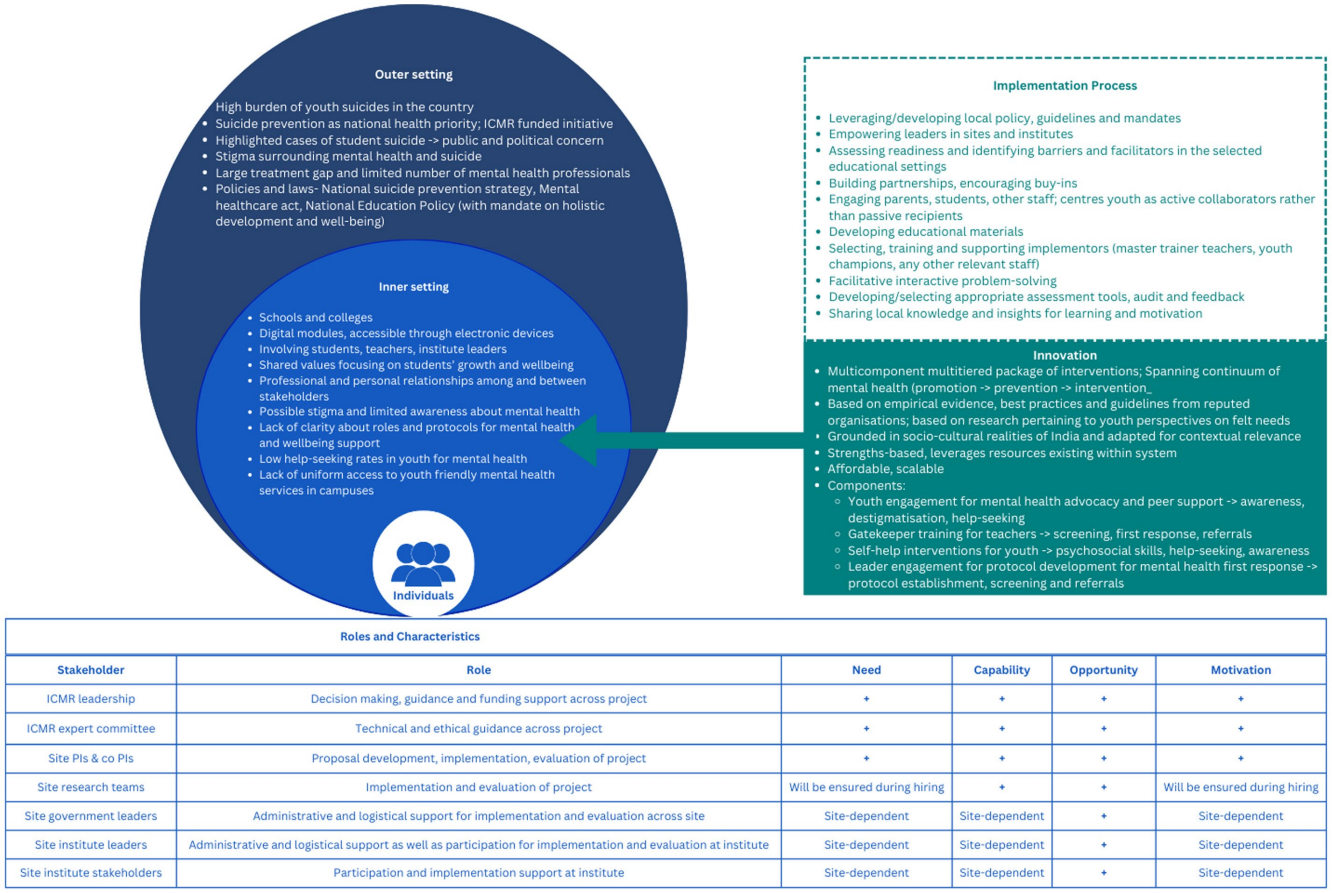
Adapted from the Updated Consolidated Framework for Implementation Research. Adapted with permission from (16), licensed under CC BY 4.0, https://doi.org/10.1186/s13012-022-01245-0.

The phase aims to systematically identify and engage key stakeholders both at state and district government departments (health and education sectors) along with professional, organizational and social networks. The primary objective of the phase is to orient stakeholders regarding rationale, scope and potential scalability of the project and to seek their commitment and participation in its implementation. The preparatory phase will involve the following activities:

1. Stakeholder mapping to develop shared understanding of the implementation project and mobilize their support in the district. This will be achieved through holding consultations with governmental (education and health) agencies and representatives; administrative heads of educational institutes and health care providers such as psychiatrists/psychologists/private physicians/government health facilities to provide referral support to distressed/high risk youth.
2. Youth awareness and sensitization for mental health issues will be done through observing mental health days; disseminating messages through posters, pamphlets and wall paintings at strategic locations in both educational institutes and health facilities and media coverage in the form of local radio and television networks. A series of orientation sessions will be conducted with all stakeholders including youth associations; students clubs in the district to influence youth behavior toward mental wellbeing and contribute as youth champions.
3. Sensitizing workshops with administrative heads of educational institutes will be conducted to present the intervention model, and planning delivery mechanism(s) and to solicit their feedback on potential implementation barriers and facilitators.
4. Capacity building workshops for healthcare professionals in collaboration with district health authorities (or under district mental health program) will also be conducted to establish referral pathways for identified distressed and at-risk students.
5. Exploring credible initiatives (NGOs, initiatives by local associations etc.) that may be operating in the region with overlapping objectives and examining scope and utility for synergy/collaborations.
6. Selection and enrolment of the implementation-clusters as planned at each site, based on willingness to participate.
7. Translations and adaptations of the resource materials and training related to the intervention components as well as preparations of the same in ready-to-implement formats.
8. Stakeholder interviews will be conducted to gain a comprehensive perspective through focus group discussions (FGDs) and in-depth interviews (IDIs) with various participants. IDIs will be conducted with administrative authorities, while FGDs would be conducted with gatekeepers and youth champions to document barriers along with enablers for implementing the intervention components and overall acceptability of the program.
9. Formative research: The initial stakeholder mapping, sensitization and capacity building workshops along with stakeholder interviews will form the situational analysis. This phase shall be of particular use to identify potential challenges and enablers in model implementation, guided by the updated Consolidated Framework for Implementation Research (CFIR) (16) (refer Figure 3).
10. Developing implementation Model 0 (M0) is derived from prior literature evidence and framework and expert consensus during the proposal development workshop at ICMR. This framework utilizes Mann Model (for selecting the targets in school/college) and Sakashita model for suicide prevention (multilevel approach, linking different types of prevention approaches) (15), suicide prevention continuum (utilizes public health interventions-primary/secondary/tertiary, capacity building, policy development) (42), and collaborative care models/framework (as informed by WHO, UMEED document, NSPS). The model aims at promoting mental wellbeing and reducing risk of suicide through increase in level of awareness, de-stigmatization, enhanced support within educational institutions for mental wellbeing, improved rates of help seeking behavior and easier access to professional care.

This phase also entails setting up of study sites with recruitment and training of staff, adaptation of resource materials and identification and seeking consent for study participation from educational institutes (4–5 clusters) for subsequent phases. The outcomes from the preparatory phase will be used for model review, redesign and refinement to enhance feasibility, efficiency and acceptability and arrive at Model M0 +.

### Phase 1 (optimization of model M0+)

The primary objective of this phase is implementation of the initial intervention model via iterative cycles.

Each study site expects at least 3–4 cycles of model optimization followed by implementation, evaluation and refinement to reach a scalable high coverage model. During each cycle, lasting 2–3 months, data will be collected on a subsample of 4–6 clusters of educational institutes in the district from leaders, gatekeepers and youth beneficiaries via in-person and online administration of questionnaires. The data shall be collected on both clinical and implementation outcomes, at baseline and at an interval of 2–3 months of model implementation. The outcomes of the phase 1 will guide in identifying the most promising model for scalability (Mref) and also entail the following:

- Adaptation of training materials: Training manuals for leaders, gatekeepers and youth shall be adapted to meet specific and contextual relevance for the selected study districts along with the development of self-help and prevention resource materials for youth.
- Establish referral support mechanism: Each study site will aim at establishment of referral mechanism for providing adequate care for students at potential high risk. This would be achieved via orienting and engagement with existing district health care service providers and galvanizing capacity building initiatives of health care professionals to respond toward high risk youth.

Following the model optimization, co-development workshops shall be organized with all key stakeholders including, site investigators, officials from state health and education department as well as representatives from the ICMR headquarters. The findings will be discussed for collaborative decision making on implementation strategies and refinement of the most contextually efficient model for scalability (Mref).

### Phase 2 [implementation and evaluation of the refined model (Mref)]

**Figure 4:**
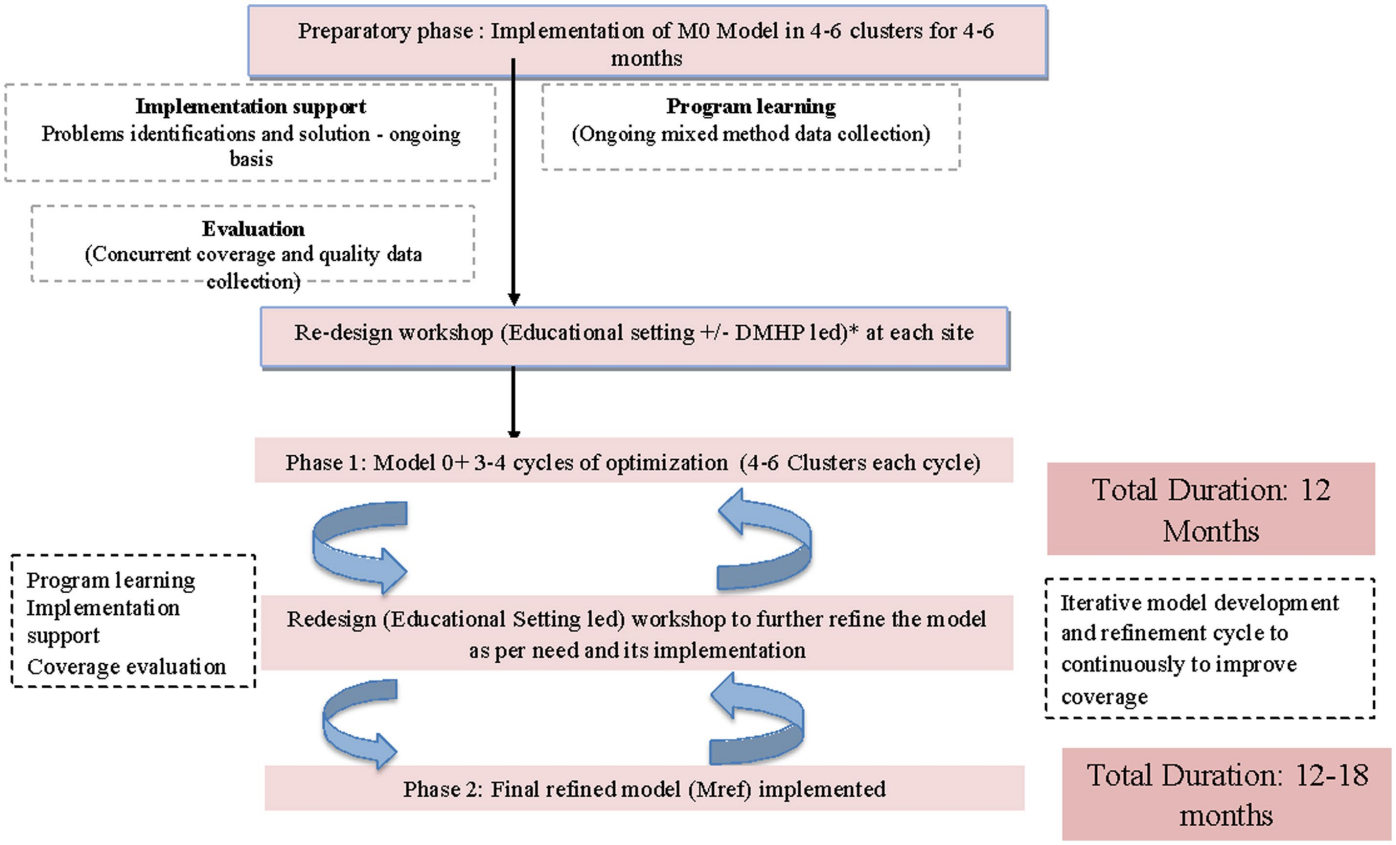
Flowchart for implementation and evaluation of Mref model.

This phase aims to implement the model (Mref) across all educational institutions over a period of 12–18 months. This shall be aimed primarily in collaboration with the district education and health authorities with research investigators mainly providing advisory and supportive roles. Guidelines, protocols and training material will be adapted to identify and train an implementation team at each institute for rolling out of the Mref model (Figure 4).

Implementation at each cluster will engage administrative authorities/heads of institutes along with volunteering gatekeepers and youth champions who will be intensively trained to implement the Mref model. Throughout phase 2, both the district education and health system will be supported to conduct capacity building of school and college administrators; teachers and youth champions and health care professionals to handle referrals of high risk students. This phase involves following key activities:

1. Expansion and engagement of resources and staff within the district education system; educational institutes and district health systems including private practitioners will be done through a series of consultative meetings with district education and health system.
2. Consultative meetings with administrative heads and district government agencies to develop standardized guidelines and protocols for management of suicide prevention at the institutes.
3. Data on implementation and clinical outcomes will continue to be collected via the research team along with conducting bi-monthly meetings with the implementation teams to provide supervision, feedback and troubleshooting implementation challenges. The selection of assessment measures has been guided by the suitability of measures for the target group and purpose, brevity of the measures, cultural appropriateness, and prior utilization in the Indian context.

#### Tools for use for gatekeepers

- Knowledge and self-efficacy survey (43): The questions of the scale are derived from a gatekeeper training survey developed by QPR institute and the research on effectiveness training (44). The four items on knowledge include question on asking directly about suicidal thoughts, understanding that they want help, knowledge on the potential to help them even when they have made suicide plans and identification of warning signs. These were selected and phrased to reflect suicide prevention facts that are most relevant to gatekeeping. The questions are answered as ‘no’ ‘maybe’ or ‘yes’. with “no” coded as 1 and “yes” coded as 3. There are 3 questions on self-efficacy which include Comfort in asking, preparedness to connect to help and confidence as to where to refer. The questions are answered on a Likert scale (4-point) varying from “not very,” “somewhat,” “very,” to “extremely.” The final self-efficacy score is obtained by adding individual item scores. In addition, an item on Intent to ask (45) will be included to assess respondents’ likelihood (i.e., intent) of asking someone who appears to be at risk or have thoughts of suicide, using a 5-point Likert-type scale.
- Gatekeeper help giving behaviors. These would include the following items using a specified time frame (last 3 months) (a) whether or not a respondent believes he/she had encountered someone who seemed to be in psychological distress (Yes/No/do not know or maybe) (45), (b) number of times they asked someone they recognized as possibly suicidal, directly/indirectly, if he or she had been thinking about suicide (45). In addition, the following additional questions on gatekeeper behaviors would be asked (43). (1) The number of individuals who approached the gatekeeper with thoughts or feelings of suicide; (2) the number of youth whom the gatekeepers themselves had approached regarding potential suicidal ideation and (3) the actions taken by gatekeepers based upon these conversations (43).

#### Tools for youth champions and youth beneficiaries

- Patient Health Questionnaire (PHQ)-9: The present study will also be using a 9-item self-report version of the Primary Care Evaluation of the Mental Disorders (PRIME-MD), a validated screening tool for identifying levels of depression among individuals (46). It is composed of 9 items to capture depressive symptoms. The total scores may range from 0 to 27 and five levels of severity varying from minimal (0–4), mild (5–9), moderate (10–14), moderately severe (15–19) and severe (20–27) (46).
- Perceived Stress Scale (PSS-10): A 10-item self-reporting screening tool for identifying level of personal stress and the degree to which an individual appraises their daily life circumstances as stress-inducing (47). The total score is the sum of all items, including reverse scoring for positive items (item 4, 5, 7 and 8). The total score ranges from 0 to 40 and three levels of severity ranging from low (0–13), moderate (14–26) and high (27–40) (47).
- Mental Health Awareness. This 19-item measure, with each item comprising three response options: false/true/do not know. ‘True’ responses to correctly worded items from this section receive a score of one, while ‘true’ responses for incorrectly worded items receive a score of zero. This is reversed for three items (items). ‘Do not know’ as a response to any item is given a score of zero. It was developed as part of a larger study on promotive intervention in college youth, in an Indian setting, via an extensive literature review, content validation by experts and 3 pilot trials covering 238 college going youth. The measure exhibited satisfactory reliability and its scores correlated positively with the measure of attitudes toward mental health and illness and showed significant changes following participation in a promotive intervention (48). Two additional items (myths/facts) pertaining to sadness vs. depression and usefulness of help seeking when distressed are planned to be included in addition (38).
- General Help Seeking Questionnaire (GHSQ): Help seeking inclination refers to the likelihood of seeking help from one or more sources for psychological distress (49). The tool assesses the participant’s intention to receive aid from varied sources as well as the inclination to reject /not seek help from any source (help negation). GHSQ is a flexible tool, allowing researchers to make modifications as per study requirement for instance, adapting sources of help and types of problems/issues to enhance relevance for the target population. Seven to twelve sources have been listed in previous studies and psychometric properties (internal consistency, convergent validity) have been found to be satisfactory across studies (50, 51). A 7-point Likert scale is used for ratings for each of the potential sources, varying from 1 (Extremely Unlikely) to 7 (Extremely likely) (49). In the present study, help seeking preferences from both formal and informal sources (e.g., teachers, counselors, helpline numbers, peer, guardian etc.) for emotional distress will be examined along with the item on help negation. In addition, an open-ended item for any other sources of help will be used to capture any unlisted source.
- Actual Help-seeking Behavior: This would be captured by enquiring about help sought within a specified time frame from one or more of the listed sources for personal/emotional problems. The list of sources would match the sources used in GHSQ. The assessment would use a checklist of items and the youth would also be given an option to briefly mention the type of issue/distress prompting the help-seeking behavior (50).
- Helping Giving Behaviors: Multiple choice questions would be used for the present study to assess if the youth encouraged someone who may have been experiencing mental health concerns to reach out for professional help (No, 1 person, 2 persons, more than 2 persons). Additionally, they would be asked if the encouragement provided actually led to the person seeking help (yes/No/not sure) (52).

#### Other tools

- Acceptability, appropriateness, feasibility of intervention measure: The implementation outcomes of the present study will assessed across three measures ‘Acceptability of Intervention Measure (AIM)’, ‘Intervention Appropriateness Measure (IAM)’, and ‘Feasibility of Intervention Measure (FIM)’ (53). The 5-point Likert scale comprises 12 items (4 items for each construct), with responses varying from ‘completely disagree’ (score 1) to ‘completely agree’ (score 5) for each item (53). This will be used with gatekeepers, youth champions and beneficiaries with a focus on ascertaining acceptability, appropriateness and feasibility of gatekeeping, conducting universal campaigns and activities and overall promotive program, respectively.
- Reach (exposure) and feedback: The youth beneficiaries would be enquired if they were exposed to and participated in any mental health promotion program event/initiative within the specified period.
- Feedback questions will be developed for gatekeepers, youth champions as well as youth beneficiaries to document overall acceptability of the program, perceived gains, if any as well as likelihood of recommending to others/participating in future.
- Semi structured interviews: Semi structured interview probes will be developed for use with leaders/coordinators (IDIs), youth champions and gatekeepers (FGDs) to document barriers as well as enablers for implementing the intervention components and overall acceptability of the program

#### Assessment time-points

The gatekeeper tools will be used at baseline (before gatekeeper training) and at 3 months intervals. In addition, all the tools except Gatekeeper help giving behaviors will also be used immediately after the training (post training assessment). Similarly, all the tools for youth champions and youth beneficiaries will be used at baseline (before the youth champion training) and subsequently at three-month intervals. In addition, these tools (except helping and help giving behaviors) will also be used immediately following the training for youth champions (post-training assessment). The general tools will be used at a three-month interval after the gatekeeper training and youth champion training are completed as described in Table 3.

**Table 3:** Description of study timelines for assessments.

Participant	Variables	T1 (Baseline)	T2 (4–6 months after T1)	T3 (12 months after T1)	T4 (18 months after T1)
Gatekeepers	Knowledge, Self-Efficacy, Intent to Ask	✓	✓	✓	✓
	Help-Giving Behaviors	✓	✓	✓	✓
Youth beneficiaries and youth champs	Depressive Symptoms (PHQ-9)	✓	✓	✓	✓
	Perceived Stress (PSS-10)	✓	✓	✓	✓
	Mental Health Awareness	✓	✓	✓	✓
	Help-Seeking Inclination (GHSQ)	✓	✓	✓	✓
	Help-Seeking Behavior	✓	✓	✓	✓
	Help Giving Behaviors	✓	✓	✓	✓
General	Acceptability, Appropriateness, Feasibility (AIM, IAM, FIM)	✗	✓	✗	✗
Exposure to program components and feedback	–	✗	✓	✓	✓
Semi-structured interviews (Barriers/Enablers)	IDIs/FGDs	✓	✓	✓	✓

### Implementation strategies

Successful implementation of any intervention program/model is dependent on appropriate and strategic use of strategies that can facilitate adoption, use and sustainment of the program. Implementation strategies target individual and contextual level mechanisms that are likely to influence implementation processes and implementation outcomes (54, 55).

The present study will be guided by the compilation of School Implementation Strategies, Translating ERIC Resources (SISTER) (56) that adopted the taxonomy of ERIC strategies (Expert Recommendations for Implementing Change) to educational settings. Examples of potentially useful SISTER strategies relevant to the context of this implementation project include leveraging policy and guidelines, empowering leaders, identifying implementation facilitators and barriers, building local partnerships, training and supporting implementers and improving buy-ins and motivation, developing educative materials, facilitating interactive problem solving, audit and feedback. The final choice and mix of strategies would be based on the local context/settings of implementation and these are also likely to be dynamically used/adapted based on continuous field observations during implementation. The strategies and processes related to implementing the intervention package for present study are entailed in Table 4.

**Table 4:** Implementation outcomes and data sources.

Implementation outcome	Institute-level measure	Youth-champ level	Teacher gatekeeper level	Data source/Method
Adoption: Intention, initial decision, or action to try or employ the intervention/uptake	Number of institutes approached. Number of Expression of interest received Number of institutes finally enrolled. Number of institutes with full implementation Number of institutes with partial implementation (implementation of 50% or more or less than 50% of intervention components)	Success of recruiting desired number of youth champions per institute depending on target number of sections/classes (Minimum 2 per class)	Success of recruiting and training desired number of teachers per institute depending on size of target population of students (30% of teachers teaching the target population)	Administrative data and records
Reach: The number and proportion of individuals who are exposed to and participate in a program	—	Number and proportion of youth champs who engage in the program The number/proportion of youth beneficiaries who are reached/exposed to the program	Number and proportion of gatekeepers who engage in the program	Responses from the target population (youth beneficiaries) regarding exposure to, participation in the intervention programs conducted, and awareness about nature of resources made accessible and how to access the same
Appropriateness: Perceived fit, relevance to address a particular issue	–	Appropriateness of campaign -based intervention	Appropriateness of campaign-based intervention and gatekeeper intervention	Intervention Appropriateness Measure (IAM)*
Feasibility: The extent to which the program can be successfully carried out within a given setting	Overall feasibility of implementing intervention package at institutes	Feasibility of campaign -based intervention	Feasibility of campaign -based intervention and gatekeeper intervention	Feasibility of Intervention measure (FIM)* (Youth champs, gatekeepers) Interviews with leaders and coordinators at institutes Field observation notes
Fidelity: Degree to which the intervention was implemented as planned	–	Sampling of universal campaigns conducted (direct observations/records of the same)	Examination of data on gatekeeper behaviors measure	Observations, Gatekeeper behaviors data and Other relevant logs/records, e.g., referrals made following identification of high distress
Acceptability: Perception of stakeholders that a given intervention is agreeable	Leaders/Coordinators’ Views	Rated by Youth champs and sample of beneficiaries: Universal programs/Campaigns	Rated by gatekeepers about gatekeeping intervention	Acceptability of Intervention measure (AIM)* plus additional feedback items Interviews with leaders and coordinators (Regarding overall package)
Implementation cost				Pragmatic approach by Cidav et al. (62) **
Effectiveness	Mean score of: Depressive symptoms-PHQ-9 Perceived stress-PSS10 Help seeking inclination-GHSQ Help-Seeking Behavior	Proportion of youth champions who were approached by distressed students for help Proportion of distressed students referred by youth champions to gatekeepers or school leaders Proportion of students who accessed self - help modules	Proportion of gatekeepers who were approached by distressed students for help Proportion of distressed students referred by gatekeepers	Administrative data and records self-reported/log books maintained by gatekeepers and youth champions
Sustainability: Extent to which the intervention is maintained/institutionalized	Key informant interviews by district authorities and leaders			Testimonials by gatekeepers and youth champions and youth beneficiaries if possible

**Described in detail under statistical analysis heading.

### Study outcomes

#### Primary outcomes

The present study focuses on the following primary outcomes:

1. Reduction in suicide risk factors: This is measured by a decrease in perceived stress levels and depressive symptoms among students in educational institutions using validated scales.
2. Improvement in help-seeking behavior: This is measured by a rise in the proportion of individuals engaging with services/aid for mental health.

#### Secondary outcomes

The expected secondary outcomes are as follows:

1. Proportion of educational institutes who started integrating the model and forming a school wellness team.
2. Incremental cost of the implementation of the model.

### Implementation outcomes

Implementation outcomes serve as indicators of success of implementation and are also seen as proximal indicators of implementation processes.

#### Implementation outcomes

Implementation outcomes serve as indicators of success of implementation and are also seen as proximal indicators of implementation processes (57). The implementation outcomes are aligned with the RE- AIM framework (17). These would capture ‘Adoption’ at setting level, ‘Reach’ and ‘Effectiveness’ at individual level, ‘Implementation’ through documenting feasibility, fidelity, appropriateness, acceptability and observations on ‘Maintenance’ of the program activities in the last phase of the project when the support from research-team is gradually withdrawn.

The implementation process will be evaluated by the COM-B model (58) of the Behavior Change Wheel (BCW). As per this model, there are 3 key factors & multiple sub-factors capable of changing behavior, namely capability, opportunity, and motivation.

Program learning shall be documented using the qualitative methods (FGD, IDI), including listing barriers and facilitators, reasons for not following through with treatment, follow-up consultations (provider plus beneficiary), feasibility and acceptability of the training module/program through questionnaires and FGDs, technical and logistical challenges in delivering the program, challenges faced by staff during the completion of the course and their subsequent engagement (reaching out and referring), difficulties encountered when participating in the online refresher training, and the overall satisfaction level of staff post-training. The timeline for all the study-related activities is provided in the Gantt chart (Table 5).

**TABLE 5:** Study timeline and Gantt chart.

Activities	Year 1												Year 2												Year 3											
Approvals - Ethical, ICMR, Govt officials																																				
Recruitment of staff, training, identifications of schools/colleges																																				
Preparatory phase (resource mapping, formative research, content development, translation)																																				
Phase 1 - Implementation and iteration cycle of model M0 + to reach refined Model (Mref)																																				
Phase 2 - Implementation of Mref model																																				
Annual progress report																																				
Final report and dissemination of results																																				

### Cost analysis of the mental health and well-being improvement intervention model

#### Conceptual framework

The implementation costs will be estimated using the pragmatic approach (59), that integrates time-driven activity-based costing (TDABC) informed by process mapping (60) and guided by an implementation science framework (54) for specifying and reporting strategies for implementation.

In the first step, process mapping will be conducted to capture detailed information on actors (who perform the activities), actions (what is done), temporality (when the activity occurs), and dosage (frequency and duration of activities) in line with the Proctor et al. framework. Subsequently, the costs of the implementation strategies will be estimated by identifying the quantity of resources required to execute the activities, and assigning appropriate prices to these resources.

The assessment of costs will be undertaken from a payer’s perspective, which reflects the direct financial expenditures incurred by the government for the implementation of the strategies. This perspective captures the additional resources required for implementing the intervention, including expenditure on personnel, equipment, etc., while excluding costs borne by patients, caregivers, or society at large. By adopting this perspective, the analysis will generate policy-relevant estimates of the financial commitments necessary for scaling and sustaining the implementation package within the existing system.

### Data collection

Data collection will be conducted in a retrospective manner to document the activities undertaken and their frequency across three phases: the pre-implementation phase (e.g., initial meetings, development of training manuals), the refinement phase (pilot testing and adaptation of the intervention), and the implementation and sustenance phase (trainings, meetings, and other activities carried out as per the refined model framework). The activities will be cost separately for each type of educational institution, considering the heterogeneity in functioning and resource use.

For each category of activity, the corresponding quantity of resources for which financial allocations were made will be assessed through stakeholder interviews with implementation teams and a review of relevant records. Resource prices will be obtained from records on actual financial allocations made, supplemented by information from personnel interviews, administrative documents or standard cost databases. For activities performed by the research team to support the implementation of the strategies, shadow pricing will be applied to document the full implementation costs to facilitate potential scale-up. In contrast, activities conducted exclusively for research and evaluation purposes will be excluded from the costing analysis. Likewise, the opportunity cost of undertaking implementation activities will not be considered.

In parallel, the evaluation will capture the impact of the suicide risk reduction strategy on healthcare-seeking behaviors, specifically by measuring the change in the number of students accessing health services following referral by educational institution staff at three time points: baseline (pre-intervention), midline (6 months), and endline (12 months). The incremental costs of outpatient consultations resulting from these referrals will also be incorporated into the final cost of the implementation package.

### Data analysis

All data will be entered and analysed in Microsoft Excel. The cost per unit of each activity will be estimated by multiplying the incremental resource use with the corresponding unit prices. The proportional distribution of resources across activities will also be assessed. Total costs will be calculated by combining the frequency of each activity with its respective per-unit cost. In addition to total and activity-specific costs, cost-effectiveness will be assessed by calculating the incremental cost per unit reduction in Patient Health Questionnaire-9 (PHQ-9) and Perceived Stress Scale-10 (PSS-10) scores.

### Data governance (management, confidentiality, security, and quality control)

Personal data of research participants will be collected but subsequently anonymized. Every participant will be assigned a unique study ID, and only anonymized data will be input into the study’s primary database on the designated analysis computers. The original identifiable information, alongside a reference sheet linking study IDs to the initial data, will be separately saved in a password-secured folder on an encrypted hard drive, ensuring maximum safety. Access to this data will be exclusive to the site principal investigators (PI) and the data manager. Anonymized data will be retained in password-secured directories on analysis computers, with only the PI, Co-Investigators (Co-Is), and specific research personnel having access rights. Primary materials such as FGD transcripts, interview recordings, and collected surveys and questionnaires will be preserved for a span of 5 years post-study, adhering to standard guidelines. A comprehensive data management guide will be crafted detailing the procedures for data handling, storage, and accessibility. Study data will be collected and managed using REDCap electronic data capture tools hosted at Indian Council of Medical Research, headquarters (62, 64). Each site investigator will be tasked with ensuring the data is updated on a weekly basis.

### Monitoring and evaluation

The monitoring and evaluation (M&E) framework uses the logical framework approach, drawing the logical link between activities and inputs to the output and outcomes, to reflect the impact. The M&E indicators will be specifically defined. The indicators are numbered measurements that help track progress on outputs and outcomes. These indicators will help trace performance but refine programs, along with building evidence for replication. The framework will enlist all indicators that will be used to assess outputs, along with frequency of data collection and source of data collection. The steps to be followed include (a) developing the objectives of the program/innovation; (b) developing the logical framework; (c) outlining indicators for evaluating output and outcomes; (d) identifying methods and tools to get data on indicators; (e) developing tools for each indicator; (f) developing a Gantt chart for all implementation phases and project activities; (g) drafting an activity monitoring checklist and decide frequency for monitoring activities; (h) baseline data collection; (i) conducting and monitoring activities during the model implementation phase; (j) recurring assessments at different time intervals; (k) analysing data and disseminating project results.

### Quality control

The national coordinating unit at ICMR headquarters will conduct monthly online meetings with study site investigators to review overall progress and address any project administration issues. In addition, weekly meetings will also be held to assess data collection and evaluations, using REDCap data quality reports to ensure data integrity and consistency. To further strengthen quality assurance, the ICMR team will also undertake on-site monitoring visits ensuring training implementation and adherence to study protocols across all study sites.

## Statistical analysis

Quantitative analyses will be conducted using the IBM SPSS, while NVivo will be used for qualitative data analysis.

### Quantitative analysis

Data analysis would be done with IBM SPSS software (version 25.0) and R software (version 4.5.1). Nominal or categorical variables would be summarized as frequency and percentages. Continuous variables will be summarized as mean and standard deviation when normally distributed and as median and interquartile range (IQR) when non-normally distributed. Frequency and percentages will be used to describe categories of coverage (full/partial/not attempted), adoption, and reach of intervention within a cluster. The coverage will be compared across different categories (school types, study sites, urban/rural) using Chi-square test or fisher exact test.

- Both clinical and implementation outcomes will be recorded as binary variables. Scores above or equal median will be considered as yes and below median as no for clinical outcomes while for implementation outcomes all the institutes will be evaluated as high performing (implemented 2 or more interventions) or low performing (implemented less than 2 interventions) based on the level of implementation. The study will employ the mixed effects logistic regression model within the generalized linear mixed models (GLMMs) framework to predict both types of binary outcomes, allowing for inclusion of both random and fixed effects. Covariates for assessing coverage of institutes will include type of institute; rural/urban location; gatekeeper to student ratio; youth champions to youth beneficiary ratio in modeling equation. For predicting help seeking behavior of students, covariates will include age and gender of student, education and occupation of parents, any known stressor in family such as recent death of close one; single parent; any past history of mental illness or suicide in family; etc.
- Time series analysis will be used for mathematical projection of the help seeking behavior of the youth beneficiaries in the study clusters. Forecasting model ARIMA (Autoregressive Integrated Moving Average) will be used to make projections till 12–18 months after the study period based on the data obtained at 3 time points (0, 6, 12 months).

### Qualitative analysis

Transcripts from FGDs and IDIs will be independently translated and transcribed. The results from the in-depth interviews and FGDs would be triangulated. Qualitative data management will be done using NVivo software, employing a combination of inductive-deductive approach. Inductive analysis will be done through reading the interview transcripts, and codes and subcodes will be generated. More precisely, thematic content analysis will be used to generate codes and sub-codes, themes and sub-themes. Results of the analysis will inform the design and development of the M0 + model, comprehensive wellbeing package. The overall framework of the intervention package and M0 + model will be finalized after the formative phase.

### Economic analysis

Microsoft Excel 2021, along with TreeAge software, is proposed for carrying out the proposed economic evaluation.

### Ethical considerations and declarations

#### Approval from the state health and education departments

Permission for conducting the study shall be obtained from the respective health and education/higher education departments of each state and union territory included in the study.

#### Ethics committee approval

The research protocol has been approved by the eight institutional ethics committees situated at Andaman & Nicobar Islands (Andaman and Nicobar Islands Institute of Medical Sciences, Sri Vijaya Puram), Arunachal Pradesh (Rajiv Gandhi University, Papum Pare), Assam [All India Institute of Medical Sciences (AIIMS), Guwahati], Karnataka [National Institute of Mental Health and Neuro Sciences (NIMHANS), Bengaluru], Kerala (Government Medical College, Kollam), Maharashtra [Tata Institute of Social Sciences (TISS), Mumbai], New Delhi [Institute of Health Behaviour and Allied Sciences (IHBAS)], and Rajasthan [All India Institute of Medical Sciences (AIIMS), Jodhpur]. The research will be carried out in alignment with the principles of the Helsinki Declaration and after obtaining clearance from the Institutional Ethics Committee. Important protocol modifications (e.g., changes to eligibility criteria, outcomes, and analyses) to relevant parties (e.g., investigators, the Institutional Ethics Committee, trial registries, journals, and regulators) will be communicated. Written informed consent will be obtained from the participants. Protocol amendments will be submitted to the respective IECs.

All self-help and training resources/material created on mental health and well-being will be checked for quality and accuracy. The material will be strengths-based, non-stigmatizing and will be evidence informed. All self-help and training resources/material and data collection tools will be translated in local languages to ensure access/reach and the translations will be reviewed for quality. In addition, the master trainers and team members conducting training for the program will be duly trained and will ensure safety of all trainee participants. The research team will also be trained and oriented to research ethics.

To safeguard the rights and interests, along with seeking permission from school leaders, an assent from the adolescent participants along with an informed consent will be obtained from their parents/guardians. For students in residential schools, permission will be sought from the warden or guardian, as it may not always be possible to reach the parents. All participants will be informed that the information they share will remain confidential and will not be shared with other students or stakeholders, with the exception of harm to themselves or others. Since the study will ascertain data about stress levels and self-harm, participants will be asked to share on the PIS/PICF the information of a trusted adult/parent who can be contacted for support if they have high distress or are at high risk for self-harm.

#### Management of adverse events

In instances where participants reports high distress or risk of harm on the study tools, the research team will inform the gatekeeper of the respective institute to assess risk and as needed follow the protocol of high-risk situations and contact the trusted adult (for participants above 18 years) or parents (for participants below 18 years) as per limits of confidentiality. The consent for this will be sought from the students at the start through the PIS and PICF and this will be clearly mentioned in the same. The team will not inquire into or insist on participants sharing any emotionally difficult material or making any personal disclosures. The research team will be trained to identify and provide immediate psychosocial support to participants expressing any emotional distress during the program. If additional support is deemed necessary, the contact details of trained counselors or helpline numbers offering subsidized/free psychotherapeutic services shall be provided to the participants and their families.

## Discussion

The protocol outlines a comprehensive, implementation model for suicide prevention among students at educational institutes across selected states in the country. The study focuses on mental health promotion, early risk identification and establishment of structured referral pathways. Varied contributing factors spanning academic stress, family issues, interpersonal conflicts, anxiety, performance pressure besides others, contribute to suicide being a leading cause of death among individuals aged 15–29 years in India. Prior literature emphasizes comorbid psychiatric conditions significantly heighten the risk of suicide among youth, underscoring the need for multi-component interventions to address overlapping mental health concerns in educational institutes (7, 61) his study aims at addressing critical gaps in the current prevention mechanisms by integrating an evidence-based capacity building for gatekeepers, youth volunteers and linking individuals to professional care within educational systems.

This multi-sectoral implementation model is grounded in the WHO’s recommendation for youth suicide prevention. The training modules for leaders, gatekeepers and peer supporters are designed to improve early identification and appropriate response toward an individual at potential risk of suicide.

Anticipated challenges include varying levels of institutional readiness, stigma and prejudice around help-seeking and need for sustained engagement from educational authorities. Mitigation strategies include iterative model refinement phases, continuous sensitization activities and collaboration with state education and health departments. Implemented in eight states across the country, the results generated in the study would be prima-facie for policy framing and contribute in value addition and refinement of the national mental health policy and other state initiated measures for mental wellbeing of students.

Furthermore, outputs from different phases of the study will be reported separately to capture evolution of intervention and facilitate scalability. The protocol holds potential to inform a sustainable, replicable model for youth suicide prevention and promotion of mental wellbeing in low-and-middle income countries like India.

The current study has certain limitations including implementation research limited to certain states and districts; in some states it may be difficult to achieve targeted sample size due to heterogeneity in the infrastructure, staff and practices and possible differences in participant profiles across sites; however, this will increase the generalizability of our findings. In addition, results may not be attributed to individual model components (some approaches may be ineffective and will be dropped, while others with success may be preserved, sometimes with modifications). The Hawthorne effect and recall bias in reporting compliance, as well as social desirability bias (within proximity of implementation support and program learning teams) are possible limitations. Furthermore, it may not be feasible to examine longer term effects in terms of sustainability once the final model is in place as the total project duration is limited to 3 years and model iterations are planned as part of implementation research in the initial phase.

The dissemination plans include publishing the results after appropriate approval, including professional, public and private avenues such as academic journals, seminars, conferences and workshops. After the data collection, we intend to publish multiple articles based on the different phases of the study.

## Conclusion

The existing protocol is a multi-site intervention implemented at educational institutes in selected states of India. The objective is to develop an implementation model for reduction of suicidal behavior specifically, aimed at reduction of levels of perceived stress and depressive symptoms while enhancing help seeking behavior among students and optimize the model through repetitive cycles of implementation, evaluation and refinement. The study will measure the cost implications of incorporating the mental health and wellbeing improvement intervention package within the education and healthcare system.

## ICMR Suicide Prevention Study Group

Aanchal, Anand – Assistant Professor, Department of Community Medicine, Andaman and Nicobar Islands Institute of Medical Sciences; Amit, Kumar – Assistant Professor, Arunachal Institute of Tribal Studies, Rajiv Gandhi University, Arunachal Pradesh; Ammu, Lukose – Assistant Professor, Department of Clinical Psychology, Loyola College of Social Sciences, Thiruvananthapuram, Kerala; Anamika, Sahu - Assistant Professor, Department of Clinical Psychology, NIMHANS Bengaluru; Ashoo, Grover – Head, Division of Delivery/ Implementation Research, Indian Council of Medical Research, headquarters, New Delhi; Chetna, Duggal – Associate Professor, School of Human Ecology, Tata Institute of Social Sciences, Mumbai; Deepak, Kumar – Professor and Head, Department of Psychiatry, IHBAS, Dilshad Garden, Delhi; Deepshikha, Prasad – Research Scientist, Division of Delivery/ Implementation Research, Indian Council of Medical Research, headquarters, New Delhi; Dharmeshwari, Lourembam – Assistant Professor, Department of Psychology, Rajiv Gandhi University, Arunachal Pradesh; Forhad, Akhter Zaman – Department of Community and Family Medicine, AIIMS, Guwahati; Girish, N Rao - Professor, Centre for Public Health, Department of Epidemiology, NIMHANS Bengaluru; Guru, S. - Associate Professor, Department of Psychiatry, NIMHANS Bengaluru; Harshal, Ramesh Salve – Additional Professor, Centre for Community Medicine, AIIMS, New Delhi; Indu, P. S. – Professor and Head, Department of Community Medicine, Government Medical College, Kollam; Kakali, Goswami – Assistant Professor, Department of Psychology, Rajiv Gandhi University, Arunachal Pradesh; Kanak, Kataria – Assistant Professor, School of Human Ecology, Tata Institute of Social Sciences, Mumbai; Krishna, Prasad M - Professor, Department of Psychiatry, NIMHANS Bengaluru; K. John, Vijay Sagar – Professor and Head, Department of Child and Adolescent Psychiatry, NIMHANS, Bengaluru; K., Thennarasu – Professor, Department of Biostatistics, NIMHANS, Bengaluru; Lijum, Nochi – Professor, Department of Economics, Rajiv Gandhi University, Arunachal Pradesh; Limalemla, Jamir – Department of Community and Family Medicine, AIIMS, Guwahati; M., Manjula – Professor, Department of Clinical Psychology, NIMHANS, Bengaluru; Naresh, Nebhinani – Professor and Head, Department of Psychiatry, AIIMS, Jodhpur; Navratan, Suthar– Associate Professor, Department of Psychiatry, AIIMS, Jodhpur; Neeti, Rustagi – Professor, Department of Community Medicine and Family Medicine, AIIMS, Jodhpur; Neha, Dahiya – Scientist D, Division of Delivery/ Research, Indian Council of Medical Research, headquarters, New Delhi; Neha, Purohit – Senior Research Officer, Department of Community Medicine & School of Public Health, Postgraduate Institute of Medical Education and Research, Chandigarh, India; Padmavathy - Senior Nursing Officer, Clinical Nursing Services, NIMHANS, Bengaluru; Pankaja Ravi, Raghav – Professor and Head, Department of Community Medicine and Family Medicine, AIIMS, Jodhpur; Panniyammakal, Jeemon – Additional Professor, Achutha Menon Centre for Health Science Studies, Sree Chitra Tirunal Institute for Medical Sciences and Technology; Philip, Rekha Rachel – Assistant Professor, Department of Community Medicine, Government Medical College, Kollam; Philip, Sharad – Assistant Professor, Department of Psychiatry, AIIMS, Guwahati; Pragya, Sharma – Director Professor, Department of Community Medicine, Maulana Azad Medical College, Delhi; Pranjal, Dey – Department of Psychiatry, AIIMS, Guwahati; Pulkit, Verma – Scientist D, Division of Informatics and Data Center, Indian Council Medical Research (ICMR), headquarters, New Delhi; P. L, Nisha – Assistant Professor, Department of Community Medicine, Government Medical College, Kollam; Rajani, Parthsarathy – Deputy Director, Directorate of Health and Family Welfare, Government of Karnataka; Rajesh- Assistant Professor, Department of Clinical Psychology, NIMHANS, Bengaluru; Rajinder K., Dhamija – Director, IHBAS, Dilshad Garden, Delhi; Ramdas, Ransing – Associate Professor, Department of Psychiatry, AIIMS, Guwahati; Ramesh Kumar, Sangwan – Scientist-C, Indian Council of Medical Research - National Institute for Implementation Research on Non-Communicable Diseases, Jodhpur; Rashmi, Agarwalla – Department of Community and Family Medicine, AIIMS, Guwahati; Ravikesh, Tripathi- Assistant Professor, Department of Clinical Psychology, NIMHANS, Bengaluru; Sampa, Sinha – Lecturer, Department of Psychiatric Social Work, IHBAS, Dilshad Garden, Delhi; Saswati, Chakraborti – Assistant Professor, Department of Psychiatric Social Work, IHBAS, Dilshad Garden, Delhi; Satabdi, Chakraborty – Additional Professor, Department of Psychiatric Social Work, IHBAS, Dilshad Garden, Delhi; Seema, Mehrotra – Professor, Department of Clinical Psychology, NIMHANS, Bengaluru; Senthil, Amudhan – Professor, Department of Epidemiology, NIMHANS, Bengaluru; Shahzadi, Malhotra – Associate Professor, Department of Clinical Psychology, IHBAS, Dilshad Garden, Delhi; Shankar, Prinja – Professor, Department of Community Medicine & School of Public Health, Postgraduate Institute of Medical Education and Research, Chandigarh, India; Shipra, Singh – Associate Professor, Department of Psychiatry, IHBAS, Dilshad Garden, Delhi; Sukanya, Ray – Assistant Professor, School of Human Ecology, Tata Institute of Social Sciences, Mumbai; S. Sagar – District Nodal Officer, District Mental Health Programme, Kollam; Tarun, Mene – Assistant Professor, Arunachal Institute of Tribal Studies, Rajiv Gandhi University, Arunachal Pradesh; T. K., Srikanth – Professor, E-Health Research Centre, International Institute of Information Technology, Bengaluru; T. V., Anilkumar – Professor and Head, Department of Psychiatry, Government Medical College, Ernakulam; Vibha, Sharma – Professor, Department of Clinical Psychology, IHBAS, Dilshad Garden, Delhi; V. Senthil, Kumar Reddi – Professor, Department of Psychiatry, NIMHANS, Bengaluru.

## Mentor group

Prashanth N, Srinivas – Director, Implementation Research Expert, Institute of Public Health, Bengaluru; Rajesh, Sagar – Professor, Department of Psychiatry, AIIMS, New Delhi; Ravindra Mohan, Pandey – A S Paintal Distinguished Scientist Chair, ICMR, NIMS, New Delhi; Somnath, Chatterjee – Ex-Director, Department of Data & Analytics, WHO; Suman, Rao – Professor, Department of Neonatology, Implementation Research Expert, St John’s Medical College, Bengaluru; Vivek, Agarwal – Professor & Head, Department of Psychiatry, King George Medical University, Lucknow; Vivek, Benegal – Ex-Professor & Head, Department of Psychiatry, NIMHANS, Bengaluru; Yatan, Pal Singh Balhara– Professor, Department of Psychiatry, AIIMS, New Delhi.
